# Usability of eHealth and Mobile Health Interventions by Young People Living With Juvenile Idiopathic Arthritis: Systematic Review

**DOI:** 10.2196/15833

**Published:** 2020-12-01

**Authors:** Sonia Butler, Dean Sculley, Derek Santos Santos, Antoni Fellas, Xavier Gironès, Davinder Singh-Grewal, Andrea Coda

**Affiliations:** 1 School of Bioscience and Pharmacy University of Newcastle Ourimbah, New South Wales Australia; 2 School of Health Sciences Queen Margaret University Edinburgh United Kingdom; 3 School of Health Sciences University of Newcastle Callaghan, New South Wales Australia; 4 Director of Research and Innovation University of Vic–Central University of Catalonia Manresa (Barcelona) Spain; 5 Department of Rheumatology Sydney Children's Hospitals Network Randwick and Westmead Sydney, New South Wales Australia; 6 Department of Rheumatology John Hunter Children’s Hospital Newcastle Australia; 7 Discipline of Child and Adolescent Health University of Sydney Sydney Australia; 8 School of Women’s and Children’s Health University of New South Wales Sydney Australia

**Keywords:** juvenile idiopathic arthritis, child, adolescence, eHealth, mHealth, systematic review, mobile phone, pain, physical activity, self-management, quality of life

## Abstract

**Background:**

Considering the changing landscape of internet use and rising ownership of digital technology by young people, new methods could be considered to improve the current model of juvenile idiopathic arthritis (JIA) management.

**Objective:**

This systematic review aims to evaluate the usability of eHealth and mobile health (mHealth) interventions currently available for young people living with JIA.

**Methods:**

The PRISMA (Preferred Reporting Items for Systematic Reviews and Meta-Analyses) guidelines were used to oversee this review. We systematically searched 15 databases for 252 potential studies; 2 authors independently screened all quantitative studies reporting the use of eHealth and mHealth interventions for young people (aged 1-18 years) diagnosed with JIA. Studies were excluded if they did not report outcome measures or were reviews, commentaries, or qualitative studies. Study methodological quality was scored using the Down and Black (modified) checklist. A narrative descriptive methodology was used to quantify the data because of heterogeneity across the studies.

**Results:**

A total of 11 studies were included in this review, reporting 7 eHealth and mHealth interventions for young people (aged 4-18 years) living with JIA, targeting health issues such as pain, health-related quality of life, physical activity, and chronic disease self-management. The usability of the interventions was facilitated through training and ongoing support. The engagement was promoted by a combination of persuasive influences, and barriers preventing adherence were removed through personal reminders and flexible program schedules to cater to JIA and non-JIA illnesses or other commonly seen activities in childhood. The feedback obtained was that most young people and their parents liked the interventions.

**Conclusions:**

The results of this review need to be considered cautiously because of the lack of rigorous testing and heterogeneity, which limits the detailed descriptions of data synthesis. Further research is needed to consider gender differences, associated costs, and the effectiveness of the interventions on health outcomes to better support young people living with JIA.

## Introduction

### Background

Juvenile idiopathic arthritis (JIA) is the most common type of arthritis in young people [1,2] with an incidence rate of 1.6 to 42.5 per 100,000, varying across different geographical locations and ethnic groups [3-6]. Symptoms include an unpredictable trajectory of joint inflammation [7], stiffness [8], pain [9], and fatigue [10] that can persist into adulthood. The active disease can impair functional ability [11,12], school attendance [13], and sleep [14], increasing the risk of poor psychosocial health [13], social isolation, reduced exam performance [15] and career prospects [16], affecting the quality of life [10,17,18]. At present, there is no definitive cure; instead, the current best practice supports timely interventions customized to manage inflammation, thus controlling pain, improving quality of life, and preventing long-term disability [18,19].

### Improving the JIA Model of Care

Responding to the needs of young people living with JIA is a challenge because of the problems in the current model of care [19,20]. A worldwide shortage of pediatric rheumatologists (PR) has limited most pediatric rheumatology services to tertiary children’s hospitals, typically based in major capital cities. Services are also based on a retrospective method of health care delivery, where appointments are made 3 to 6 months in advance, which is slow to react to a patient’s changing conditions [20-25].

Feedback from parent and carer surveys also suggests that pediatric rheumatology services need to improve the quality of their service and the patients’ experience. Responses suggest they need to optimize their efficiency, improve information exchange [26,27], promote ongoing interdisciplinary support networks [26-28], and improve access to a JIA experienced clinician when needing urgent advice [27] or experiencing an unpredictable flare of disease, complications, adverse reactions [26], or illness [28].

### Improving Self-Management Behavior

Good self-management behavior has a positive effect on health outcomes [29]. Young people with JIA and their families need to be encouraged to take an active role in their disease management [30] and be provided with meaningful opportunities to develop the skills they need to support self-management [29]. This is important because young people do not automatically develop these skills [31], and they are not overly concerned about their long-term health outcomes. Instead, they are more concerned with the present [32], making disease management secondary to their school and social activities [33].

### Digital Solutions

An innovative way to improve the current care model and foster self-management skills could be through eHealth or mobile health (mHealth) interventions [34-36], in particular, when considering the changing landscape of internet usage [37] and rising ownership of digital technology by young people [38]. A growing generation of digital natives is inadvertently turning to digital solutions to support their daily lives [34,39]. From a young person’s viewpoint, digital technology can promote a better understanding of their disease, support self-management, and remove the need for constant supervision by parents and clinicians [36]. From a clinician’s perspective, digital technology can facilitate health promotion and allow real-time symptom monitoring [34,36], potentially enabling timely changes to treatments and the prevention of flare-ups, thereby improving health outcomes and decreasing health care costs.

Critical to the success of any digital intervention is the manner in which young people accept and interact with the technology [38,40,41]. This understanding is often uncovered in usability testing. In recent years, usability testing has shifted from traditional technology testing to understanding and optimizing the users’ experience [41-43] because user feedback can be different from the planned use of the intervention [43]. Usability testing that pursues a user-led design [35,41], particularly for specific populations, uncovers problems related to acceptability, perceived level of usefulness, and adherence. Aiding the delivery, uptake, and retention of an accessible intervention that fits into a young person’s lifestyle and meets the needs of a wide and diverse range of users [44-47].

### Definition of eHealth and mHealth

eHealth is described by the World Health Organization (WHO) as an activity that delivers health-related information, resources, and services through electronic technology and internet connectivity [48]. mHealth is described as a mobile and wireless form of technology for medical and public health practices [41].

### Aim and Rationale

This systematic review presents the first of 2 steps in evaluating the clinical use of eHealth and mHealth interventions for young people (aged 1-18 years) living with JIA. This review aims to evaluate quantitative studies examining the usability of eHealth and mHealth interventions to understand how young people interact with the technology. The following 3 areas were considered:

1. Identification of the digital health intervention.
2. Usability (delivery of the intervention) [47,48].
3. Costs associated with the intervention [48].

It is anticipated that such information will improve our understanding of the mechanisms that support the use of these interventions by young people living with JIA and inform future development. The second step of this review aims to evaluate the effectiveness of interventions. These results will be published subsequently in another review.

## Methods

### Overview

The PRISMA (Preferred Reporting Items for Systematic Reviews and Meta-Analyses) statement [49] guided this review (Multimedia Appendix 1). The protocol for this systematic review was registered on PROSPERO (International Prospective Register of Systematic Reviews; ID CRD42018108985) [50].

### Eligibility Criteria

#### Participants

All young people (aged 1-18 years) diagnosed with JIA using the International League of Associations for Rheumatology criteria [51] were considered eligible.

#### Interventions

Any eHealth or mHealth interventions (see definition in the *Introduction*) delivered through an electronic device with internet connectivity [46] or wireless capacity were eligible [52].

#### Comparator/Control

No comparator was used.

#### Outcomes

We considered an outcome as any quantifiable measure specifically targeting the pediatric population or pediatric rheumatology.

#### Study Design

All quantitative studies reporting the use of eHealth and mHealth interventions for young people (aged 1-18 years) diagnosed with JIA were included. Studies were excluded if they did not report outcome measures or were reviews, commentaries, or qualitative studies.

### Search Strategy

To develop search terms, MEDLINE and CINAHL were initially searched by SB to identify keywords in the titles, abstracts, and indexed terms. In October 2018, the search terms were adapted to suit the controlled vocabulary, Boolean operators, truncation, and wildcards in MEDLINE/PubMed, the Cochrane Library, Joanna Briggs Institute, AMED (Allied and Complementary Medicine Database), CINAHL complete, EMBASE, JAMA (Journal of the American Medical Association), Informit Health, ProQuest database, PsycINFO, IEEE (Institute of Electrical and Electronics Engineers and Institution of Engineering and Technology) Xplore, SAGE Publishing, ScienceDirect, Scopus, and Web of Science. Further studies were retrieved from Google Scholar and arthritis-related organizations (Arthritis Australia, Arthritis Foundation, and Childhood Arthritis and Rheumatology Research Alliance funded projects and conferences) and by hand searching reference lists. The search strategy was not restricted by language or year of publication. The database search was repeated in November 2019 (Multimedia Appendix 2).

### Study Selection

Two authors (SB and AC) independently reviewed all studies retrieved by the search strategy via individual log-in systems on the web-based platform Covidence [53]. Titles and abstracts were reviewed against the inclusion and exclusion criteria and full-text versions. Authorship and results were not masked, and any disagreements were discussed and resolved by SB and AC. To gain access to all full-text studies, corresponding authors were contacted by email, or the full text was retrieved by the University of Newcastle library interlibrary request service *Get It*. There was no need for translation sources; only 1 study was retrieved in a language other than English (Dutch), and an English version of the same study was attained through ResearchGate.

### Data Collection

A data extraction Excel (Microsoft) form was designed to collect all relevant information from the studies, including participant demographics, eHealth or mHealth characteristics, study design, study outcomes, and costs. Conclusions were drawn from the outcomes reported by study authors. Data extraction was completed by SB and checked by all reviewers.

### Risk of Bias

Using the Down and Black (modified) checklist for randomized and nonrandomized studies [54,55], studies were rated independently by 2 reviewers (SB and AF). This checklist has a high correlation with similar tools for validity (*r*=0.90) and reliability (*r*=0.69-0.90) [54,55]. The checklist considers 5 main assessment areas: (1) reporting; (2) external validity; (3) internal validity, bias; (4) internal validity, cofounding and selection bias; and (5) power; it provides an overall score out of 28 [56]. Across the studies, a disagreement rate of only 7.6% arose (13 of 170 questions), which was resolved through discussion (SB and AF) and re-examination of the studies.

### Summary Measures

To summarize the participants’ characteristics, the mean scores reported by the study authors were averaged. The range of data was determined by the reported highest and lowest values. Individual JIA subtypes were combined and expressed as the total number (n) and the proportion of each subtype as a percentage (%). The total number of studies including the information required was stated (ie, “4 studies reported…”) to account for missing data.

### Synthesis of Results

Meta-analysis was considered unsuitable for this systematic review because of the heterogeneity across the studies and the different intervention development stages. Instead, a narrative synthesis methodology was used to allow the data to be organized, explored, and presented in a logical way [57] to uncover potential similarities and differences, associations, and patterns within the results [57,58]. The 4 stages of analysis suggested by Popay et al [58] were adapted and used to guide this review.

1. Develop a theoretical model to understand how the intervention works.
2. Conduct a preliminary synthesis to:
  identify factors supporting implementation and barriers;
  consider relationships among studies.
3. Perform a content analysis (translation of data) to:
  report characteristics among studies;
  identify moderator variables;
  develop numerical/statistical rubrics.
4. Draw a conclusion by critically reflecting on methodology synthesis.

## Results

### Study Selection Process

**Figure 1:**
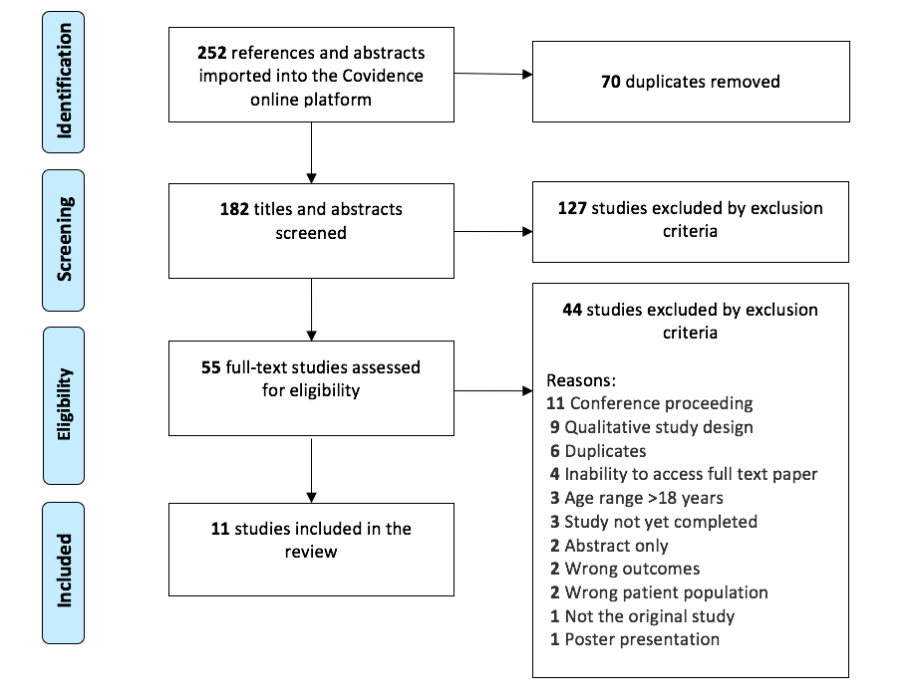
Summary of the study selection process using the PRISMA (Preferred Reporting Items for Systematic Reviews and Meta-Analyses) diagram.

A total of 252 studies were identified using the search strategy. After removing 70 duplicates, 127 studies that did not meet the inclusion criteria based on their title or abstract and 44 based on the full-text screening, a total of 11 studies met the inclusion criteria for this review (Figure 1).

### Study Characteristics

#### Participants

This review included 634 participants [59-67]; 57.1% (362/634) of participants were recruited from either pediatric rheumatology departments or clinics affiliated to a hospital, and 42.9% (272/634) of participants from pediatric tertiary care centers. Study sample sizes ranged from 13 to 176 participants and varied in age (mean 12 years, SD 2.5; range 4-18.6 years), gender (female: 429/602, 71.3%; mean 42.9, SD 31.6), and JIA subtypes (Table 1) [59-67]. To avoid duplication, a total of 2 studies were excluded from this analysis [68,69], because the participant characteristics were reported in another study included in this review [61,67]; furthermore, 3 studies did not report participants characteristics when participants were lost in follow-up (32/634, 5.0%), reducing the age and gender analysis to 602 of 634 participants [60,61,63].

**Table 1:** Juvenile idiopathic arthritis subtypes, based on the International League of Associations for Rheumatology criteria.

Juvenile idiopathic arthritis subtypes	Value
Oligoarthritis^a^, n (%)	195 (30.8)
Polyarthritis^b^, n (%)	172 (27.1)
Polyarthritis (rheumatoid factor positive), n (%)	41 (6)
Enthesitis related, n (%)	54 (8)
Systemic, n (%)	46 (7)
Psoriatic, n (%)	33 (5)
Undifferentiated, n (%)	7 (1)
Unknown or not yet diagnosed or other, n (%)	39 (6)
Chronic arthritis with other/or other forms of rheumatic disease^c^, n (%)	13 (2)
Not recorded, n (%)^d^	34 (5)
Disease activity (cm), mean (range)^e^	1.8 (0.2-3.7)
Disease duration, mean (range)^f^	5 years (<1 month-15.65 years)

^a^Includes the subcategories of oligoarthritis: persistent and extended [60,62-64,66].

^b^Includes 2 studies not reporting positive or negative rheumatoid factor. [63,67].

^c^Juvenile dermatomyositis, systemic lupus erythematosus [62,63].

^d^Reasons: drop-out, loss of follow-up (n=32) [60,61,64] and missing subtypes (n=2) [64]

^e^A total of 8 studies reported disease activity [59,60,62,63,65-68].

^f^A total of 7 studies reported disease duration [60,62,64-68].

#### Intervention

The 11 studies included in this review reported 7 interventions, describing varying stages of development (preprototype to maturity); 4 interventions were web-based programs [61-65,68], 1 intervention was a computer-mediated electronic peer mentoring program (e-mentoring) [60], and 2 interventions used mobile technology for real-time monitoring [66,67,69]. The clinical significance of interventions aimed at improving self-management behavior [59-61,64,65,68] or supporting clinical decisions [62,66,67,69]. These interventions included the following:

1. *Misfit Flash*, a commercially available wearable tracker to improve physical activity [59].
2. *Rheumates@Work*, a web-based educational and cognitive behavioral program to improve self-management and physical activity [61,64,68].
3. *iPeer2Peer Program*, an online peer mentoring program to facilitate positive role modeling and social support through video calls [60].
4. *eOuch*, a customized electronic pain diary to self-report real-time pain [66,67,69].
5. *ePROfile* a web-based assessment (Kwaliteit van leven in kaart, or, quality of life map [KLIK] website) to self-report health-related quality of life (HRQoL) issues [62].
6. *SUPER*-*KIDZ*, a web-based pain assessment to self-report real-time pain [63].
7. *Teens Taking Charge: Managing Arthritis Online*, a web-based program to provide disease-specific information and self-management strategies [65].

Custom-designed programs designed by the research team were used in 5 interventions [61-69], and 2 interventions were commercially available [59,60]. The equipment necessary to operate these interventions included computers, laptops, handheld PDAs, an Apple iPod touch, and a wearable accelerometer synchronized to a smartphone. This allowed the interventions to be used at the participants’ home [59-62,64-69] or clinic [62,63] (Multimedia Appendix 3).

#### Outcomes

Study outcomes considered feasibility, usability, efficiency, and/or effectiveness. To align usability outcomes with research questions 2 and 3, usability outcomes were synthesized to form 4 themes: (1) user engagement (promotional activities and adherence), (2) barriers preventing usability (personal, technical, and device barriers), (3) user feedback (acceptability and satisfaction), and (4) cost assessment (basic financial costs). Themes were based on 5 of 16 areas of reporting by Agarwal et al [41] to improve the quality of evidence being extracted. The remaining areas of reporting are beyond the scope of this review (Table 2).

**Table 2:** Formation of themes, evaluation criteria, and main outcomes supporting the delivery of the eHealth and mobile health interventions for juvenile idiopathic arthritis.

Research question, theme, and evaluation criteria			Outcomes
**Research question 2**			
	**User engagement**		
		Promotional activities	Adoption inputs [59-61,64-69]
		Adherence	Activity log, dropout [59] participation rate [68], program adherence [60,64], commitment, level of interaction [61], program compliance [65-67,69], and safety [59,60,64]
	**Barriers preventing usability**		
		Personal barriers, technical barriers, or device barriers	Device malfunction rate [59], barriers preventing engagement [59], technical problems [61,63,66], missed responses [63], or errors [67]
	**User feedback**		
		Acceptability	Questionaries [60,63,65] and evaluation questionnaire [66,67]
		Satisfaction	Questionaries [60-63,65], semistructured phone interview [60], and evaluation of use [62]
**Research question 3**			
	**Cost assessment**		
		Basic financial cost (owing to no comparator)	Cost (adding together development of the program, staff costs, financial consequences ie, traveling expenses and babysitters) [61]

#### Study Design

The study design included 4 descriptive studies, 3 pilot randomized controlled trials (RCTs), 1 multicenter RCT, 1 pre- and postintervention design, 1 correlational research design, and 1 sequential cohort study—studies reported from 2 high-infrastructure countries, Canada [59,60,63,65-67,69] and the Netherlands [61,62,64,68] (Multimedia Appendix 4).

### Methodological Quality of Studies

The methodological quality of study scores ranged from 15 to 21 out of 28 (mean score 18.6, SD 1.9), a fair to good score [54]. Convenience sampling and/or selection bias meant that study participants may not fully represent the JIA population. Participants were selected according to disease activity, pain, the unlikelihood of medication changes, level of physical activity, owning a computer/tablet/interactive mobile phone, availability of the internet, and literacy levels ([59-69]) Multimedia Appendix 5.

### Results of Studies-Delivery of the Intervention

#### Theme 1: User Engagement

##### Promotional Activities

All studies reported promotional activities to support engagement with the intervention. The top 2 included training [59-63,65] and ongoing human communication [60,61,64-66] (Table 3).

**Table 3:** Promotional activities used to support engagement with the intervention for juvenile idiopathic arthritis.

Promotional activity	Misfit flash	Rheumates@Work	ePROfile	iPeer2Peer	eOuch	SUPER-KIDZ	Teens taking charge
Training	Y^a^	Y	Y	Y	Y	Y	Y
Instruction manual	Y	—^b^	—	—	Y	—	—
Goals set by users	Y	Y	—	—	—	—	Y
Ongoing technical support	—	Y	—	—	Y	—	Y
Personal reminders	—	—	—	—	Y	—	—
Ongoing human communication	—	Y	—	Y	Y	—	Y
Reactive feedback loop	—	Y	—	—		—	—
Linear design	—	Y	—	—	Y	—	Y
Interactive content	—	Y	—	—		—	Y
Flexibility in length of the program	—	Y	—	Y		—	Y

^a^Y: yes.

^b^The promotional activity listed was not used.

##### Training

All interventions provided participants and/or parents with training (range <5-20 min) [59-63]. Training sessions included how to use the software [69], functionality [59,69], demonstration and practice using pain vignettes [66,69], and instruction on completing learning modules and pain entries [61,66]; 3 interventions also provided training for those supporting the intervention: PRs, peer mentors, and a coach [60,62,65]. One study reported flexibility in training, delivering training at the participant’s home or clinic [67] (Multimedia Appendix 2).

##### Ongoing Human Communication

Throughout the study period, 4 interventions provided ongoing human communication [60,61,64-66,68]. Human communication included telephone support [60,61,64-66], emails [60,64,65], face-to-face group sessions [64,68], daily monitoring of discussion boards [60,65], peer support [60, 65], and an online chat room [61]. A good-quality study, *Teens taking charge: managing arthritis online*, considered the use of a coach, providing weekly telephone calls as a necessary part of the intervention, reporting 1.6 calls (mean duration 17.3 min, range 7-30 min) each week for the intervention group (IG) [65]. After the study period, the control group (CG) was given access to the intervention, without the coach and telephone support. Website engagement dropped compared with the IG, from 436.9 to 6.42 hours. Similarly, interaction by parents of the CG reduced from 458 to 19 hits [65].

##### Safety Support

A total of 3 fair to good–quality studies monitored participants’ safety [59,60,64]. The *iPeer2Peer Program* reported that all Skype calls were recorded and reviewed within 24 hours by a research team member. The peer mentor was also trained to flag concerns [60]. For *Rheumates@Work*, the PR maintained disease activity and medication usage records, reporting disease flare-ups for 3 participants (IG, n=1/17; CG, n=2/16), and no adverse events [64]. *Misfit Flash*, reported illness, injury, or pain for 9 participants due to being active (n=9/28) and arthritis-related pain for 1 participant (n=1/28). However, no significant difference was reported for pain, functionality, or disease activity during the study period [59].

#### Adherence Rates

All interventions had an expected level of engagement, ranging from minutes to 17 weeks [59,60,62-69]. A total of 8 fair to good–quality studies monitored adherence rates (range 70%-82.1%) [59-61,63-67,69]; 4 studies increased adherence by allowing more time to complete the intervention [60,61,64,65], and 1 study increased adherence to 100% (n=46) [65] ([59-61,64-67,69] Multimedia Appendix 6).

##### Week to Week and Time of Day Adherence

A significant difference was seen in adherence in pain reporting, using the eOuch pain diary, by 2 fair to good quality studies, week to week and according to the time of day (Multimedia Appendix 6) [66,67]. Adherence rates were increased 10% across the day by adjusting the preset pain reporting reminder alarms (morning, on waking, after school, and evening, before bed) according to age, 1.5 hours later on weekend mornings for older participants and 30 min earlier in the evenings for younger participants [66].

##### Gender and/or Age Adherence

The impact of gender and/or age on adherence was considered by 2 high-quality studies [60,67]. *eOuch* (n=112) reported gender or age had no effect [67]. Conversely, the *iPeer2Peer Program* reported that male participants (n=2/18) had lower adherence [60]. The 2 male participants completed 5 and 7 video calls, respectively, instead of the expected 10 as per protocol, and call length was nearly half that of female participants (12/16) [60].

##### Content Adherence

The most common topics raised/learning modules visited by participants in self-management programs were reported by 3 high-quality studies. The most common were *understanding arthritis* and *management issues* [60,61,65] (Multimedia Appendix 6).

#### Theme 2: Barriers Preventing Usability

##### Personal Barriers

Personal barriers preventing adherence were reported qualitatively by 6 fair to good–quality studies. The main barrier was illness, both JIA- and non-JIA-related [59-61,65,68]. In the *iPeer2Peer Program,* illness affected both the participants and peer mentors [60]. Other barriers included hospitalization [61,65], injury and pain [59], and common childhood activities such as study time/assessments [59,60,65,66,69], school trips [61], extracurricular activities [59], holidays [61,66], a party, a sports tournament [61], loss of mobile phone privileges, loss of activity tracker [59], death in the family, being too busy, weather, no babysitter for siblings [61], no longer interested [61,68], and no specific reason [61,64,68].

##### Technical Barriers

Technical barriers preventing adherence were reported by 7 fair to good–quality studies [59,61,63,65-67]. Barriers included log-in [61,65], software [59,66,67], hardware [59], device [59,66], and network problems [63,66,67]. This resulted in participants dropping out of the study [65] and lost data [63,67]. Future problems were eliminated through software changes, consultation with network providers, instructions on how to reset the device [66], and data back-up [66,67] ([59,61,63,65-67,69] Multimedia Appendix 7).

##### Device Barriers

Overall, 3 fair to good–quality studies compared electronic *eOuch* pain diary entries [66,67,69] with the paper-based pain assessment—brief pain inventory (BPI), short-form [70]. In 1 study, participants made no errors using *eOuch* compared with 90.8% (69/76) of errors using the BPI [67]. Most errors were related to how they marked the visual analog scale—77% (55/76) were confused by the order of least, average, and worst pain ratings. Participants with higher pain levels made more errors. There was no statistical difference in age (*P*=.51) or sex (*P*=.40) [67].

In another fair-quality study, 3 different devices were compared by children (aged 4-7 years), adolescents (aged 8-18 years), and parents when completing the web-based pain assessment *SUPER-KIDZ* [63]. These devices included paper, a handheld Apple iPod touch (second generation), and a computer/laptop. The study reported a significant difference in the number of missed responses by adolescents using the iPod (*P*=.047) compared with parents (*P*=.16) and children (*P*=.37) [63]. The iPod also required the most amount of time for adolescents and parents (*P*<.001), followed by computer (*P*<.001) and paper (*P*<.001). There was no significant difference in the device used by children completing a 2-item survey (*P*=.64) [63].

In the same study, children preferred the computer to paper or iPod because it was simple and fun to use (*P*=.008) [63]. Adolescents least liked the iPod because of size, unfamiliarity, and increased time to complete responses *(P*=.001). Adolescents also described paper assessments as the most inappropriate for their age group (*P*=.004) [63].

#### Theme 3: User Feedback

##### Acceptability and Satisfaction

All 7 interventions gained positive feedback from young people [59-63,65-67]. A total of 8 fair to good–quality studies reported that young people liked being physically active [59], making new friends [61], meeting someone with JIA whom they could relate to, or who had already experienced what they were going through (JIA- and non-JIA related) [60], how the intervention looked [66,67], the intervention’s content [65], getting information about JIA [60], personalization through interactive features [65], and an email character called Buddy [61]. Four studies reported that young people would continue to use or recommend interventions [59,65-67]. Improvements were also suggested in exercise programs and the age range of the content [61].

Parental feedback was also considered by 2 interventions [61-63]. *Rheumates@Work*, a good-quality study, reported that parents liked the interventions (63/64, 99%) [61] and learned something (48/64, 75%) [61]. Parents also provided high evaluation scores (median 8/10, range 4-10) for *ePROfile*, reporting *ePROfile* as useful (t1=57/65, 88%; t2=37/46,80%); however, the satisfaction of parents and young people did not differ between IG and CG [62] ([59-63,65-67] Multimedia Appendix 8).

#### Theme 4: Cost of Delivering the Intervention

Only 1 of the 7 interventions reported the cost of establishing and maintaining the intervention [61]. *Rheumates@Work*, a good-quality study, reported on program content €10,000 (US $ 11,888); web design, language adaption for young people €1500 (US $ 1783); staff numbers (n=1-2 part-time and/or physician/psychologist) for facilitating group sessions; the time needed to monitor the participants’ progress and sending emails (30 min/week); and the participants’ time (1 hour/week) [61].

Overall, 4 studies reported that they provided participants with a device to enable access to the intervention [59,66,67,69]; 3 fair to good–quality studies supplied a PDA [66,67,69], and 1 fair-quality study provided a wearable accelerometer [59]. All devices were to be returned at the conclusion of the study. Only half of the participants (15/28, 55%) returned the wearable accelerometer [59]. These studies did not report on these costs, and a request for further information was unsuccessful.

## Discussion

### Principal Findings

To the best of our knowledge, this is the first systematic review to evaluate the usability of eHealth and mHealth interventions targeting young people living with JIA (aged 4-18 years). Guided by our 3 research questions, this review identified 7 interventions: *Misfit Flash*, *Rheumates@Work*, *iPeer2Peer Program*, *eOuch*, *ePROfile*, *SUPER-KIDZ*, *and Teens taking charge: managing arthritis online*. The methodological quality of the studies supporting these interventions ranged from fair [59,62,63,66,69] to good [60,61,64,65,67,68]. The dropout rate across 9 studies was low (49/634, 7.7%; mean 5, SD 6.1) [59-67].

### Identification of the Digital Health Interventions

#### Interventions to Improve JIA Model of Care

Of the interventions, 3 allowed participants to self-report pain [63,66,67,69] or HRQoL [62]. One intervention aimed to generate a computer-based pain summary [63] Another, to improve HRQoL communication during the PR consultation [62]. This form of real-time data collection has the potential to improve data processing [71] and patient monitoring, allowing well-informed, person-centered health care decisions to be made [72].

#### Interventions to Improve Self-Managing Behavior

In total, 3 interventions aimed to improve self-management behavior [61,65,68] and 2 interventions aimed to improve physical activity [59,64,68]. Participants focused their educational needs on understanding arthritis and disease management issues [60,61,65]. Understanding these needs from a young person’s perspective is important because there is often a difference in opinion by young people, parents, and health professionals to what self-management programs should include [34]. Research shows that by correctly strengthening a young person’s personal knowledge, their motivation and competence to make well-informed health decisions improve [73], reducing their long-term health risks [29].

### Usability

#### User Engagement

A range of promotional activities was used to facilitate the engagement of the participants with the interventions. These promotional activities are referred to in the literature as persuasive influences [74,75]. Although the studies in this review did not evaluate their effectiveness, notably other systematic reviews have reported their importance [74-76] and supported their inclusion in the intervention design to promote adherence [74]. In fact, for web-based health interventions, a combination should be used, for example, tailored interactive health information, reminders, and incentives to promote active engagement [75], and weekly website updates to increase log-ins [76].

The interventions in this review used, on average, 4 persuasive influences (range 1-7), the 2 most common being training [59-63,65] and ongoing human communication [60,61,64-66]. Other studies support the use of human communication through face-to-face segments, peer-to-peer support, a health professional, or counseling to increase website usage [74-76]. In this review, telephone contact was the most common type of human communication. *Teens taking charge: managing arthritis online* reported how the use of a coach providing telephone support improved website engagement [65]. Interestingly, this study did not employ a health professional, as other systematic reviews have suggested [75,76]. Instead, an undergraduate student studying psychology followed a standardized script to review homework and goals [65], possibly reducing the cost of the intervention.

However, not all forms of human communication identified in this review were supported*.* A systematic review identified 9 studies where discussion boards only provided a moderate level of peer support for young people [75]. Similarly*, Rheumates@Work* reported low engagement rates with chat sessions (17/64, 27%) [61], reinforcing the need to include young people in the design and development.

In this review, to improve the young person’s experience and ensure that the interventions were achieving the intended interactions, personal barriers were removed [59-61,63-68]. Pain diary reporting times were adjusted according to age, and program schedules were flexible to cater for JIA- and non-JIA-related illnesses [59-61,65], school, and social activities [59-61,65,66]. Technical barriers were also overcome by most interventions that store their data on an external server, rather than the electronic device being used [60-62,65,66]. The privacy of health information was further maintained through secure participant accounts with restricted access [61-63,65,66]. For example, the PR could only see their own patients’ results [62]. Similar measures are reflected in other studies, supporting the use of certified servers and data security, despite being costly and requiring a technical team to set up and maintain it [77,78].

#### User Feedback

All interventions in this review gained positive feedback from young people [59-63,65-67] and parents [61-63], although some improvements were identified [60,61,65]. *Rheumates@Work* participants, for example, requested more specific exercise programs and age-appropriate content. The targeted age range of 8 to 13 years was too broad—slightly difficult for younger participants, and too childish for older participants [61]. The transparency of the intervention content is also important because the WHO suggests that content needs to align with national guidelines or regulatory statutes; if the intervention is successful, it may be considered as a medical device [41]. Only 4 interventions in this review aligned their content with either guidelines or regulations [61,64], validated tools [62,66,67,69], a research methodology (Delphi technique) [63], and/or learning theory [61,64].

### Cost Associated With the Intervention

Only 1 study in this review reported costs related to the implementation of their intervention [61], despite the WHO strongly suggesting this [48]. Costs should include long-term direct and indirect costs, starting from software development to training, implementation, and the end benefits for patients and the health care system [79]. For example, a recent systematic review of the utilization of mHealth interventions reported reduced travel time and fuel costs for health care workers and patients, and increased working time for health care workers [80]. Considering costs early, during prototype development, may help inform strategic decisions to ensure the intervention, if successful, is cost-effective, easily accessible, and sustainable when translated into the community [79].

### Future Research to Consider Gender Differences

Only 3 studies in this review considered gender differences [59,60,67]. This is probably because the JIA population is predominantly female, with 3 to 6.6 females to every 1 male [81]. Although the findings were not significant, gender differences have been reported on internet use by men, women [82], and college students [83]. This indicates the need to include gender differences in future research to identify different support needs and/or gender-specific persuasive influences that could be adopted to promote adherence for young people.

### Limitations

The findings of this systematic review need to be considered cautiously because of the limited number of studies included. Our methodology could have been improved by including qualitative studies in our selection criteria and individually handing searching journals relevant to digital health to ensure no usability issues were omitted and reduce publication bias from the database search.

Meta-analysis was also not possible in this review because all interventions considered different outcomes measures, depending on their stage of development. Instead, this systematic review largely relied on descriptive summaries to organize and clarify the data from formal and informal assessments. This form of analysis can be subjective; participants may have been influenced by the novelty of the intervention, boosting their engagement and feedback. There is also a risk of reporting bias by the author. To reduce this risk and improve transparency, all authors reviewed each stage of the data analysis.

The generalizability of our findings may also be limited. Participants were included in this review with other forms of rheumatic disease, or their diagnosis was unknown or not recorded (86/634, 13.6%; Table 1) [62,63,66,67]. Dissecting the results for young people, specifically living with JIA, was not possible.

This review also only focused on 3 of our 4 protocol questions: (1) identification of the intervention, (2) usability, and (4) cost, rather than (3) effectiveness, to allow us to capture a more detailed description of the interventions and usability problems faced by participants. The effectiveness of the interventions will be covered in a follow-up publication.

### Conclusions

Using a narrative, descriptive methodology, our review identified 7 interventions for JIA, targeting health issues such as pain, HRQoL, physical activity, and chronic disease self-management. The usability of the interventions was facilitated through training and ongoing human communication. Engagement was promoted by a combination of persuasive influences, and barriers preventing adherence were removed through personal reminders and flexible program schedules to cater to JIA- and non-JIA illness or other activities commonly seen in childhood. The feedback obtained was that most young people and their parents liked the interventions. Although too premature to support the effectiveness of our claims, this review will add to the growing body of evidence influencing the development of future eHealth and mHealth interventions. Further research is needed to consider gender differences, associated costs, and the effectiveness of interventions on health outcomes to better support young people living with JIA.

## Appendix

Multimedia Appendix 1 PRISMA (Preferred Reporting Items for Systematic Reviews and Meta-Analyses) checklist.

Multimedia Appendix 2 Search terms and database search strategy.

Multimedia Appendix 3 Overview of the seven eHealth and mHealth interventions for JIA.

Multimedia Appendix 4 Overview of the eleven eHealth and mHealth studies targeting Juvenile Idiopathic Arthritis.

Multimedia Appendix 5 Methodological scores of the eleven studies using the Down and Black.

Multimedia Appendix 6 Intervention adherence rates, including week to week, time of day and content adherence by young people with JIA.

Multimedia Appendix 7 Summary of the technical problems experienced by users with JIA, obstructing the adoption of the intervention.

Multimedia Appendix 8 User feedback: Results of Acceptability and Satisfaction questionnaires.

## Abbreviations

BPI: brief pain inventory
CG: control group
HRQoL: health-related quality of life
IG: intervention group
JIA: juvenile idiopathic arthritis
mHealth: mobile health
PDA: personal digital assistant
PR: pediatric rheumatologists
RCT: randomized controlled trial
WHO: World Health Organization
