# Supplementary material for: Usability of eHealth and Mobile Health Interventions by Young People Living With Juvenile Idiopathic Arthritis: Systematic Review
Source: JMIR Pediatr Parent. 2020 Dec 1;3(2):e15833. doi: 10.2196/15833 (PMC7738264; doi:10.2196/15833)

## **Search terms**

1. randomi#ed controlled trials OR controlled clinical trial OR random# OR placebo OR trial OR groups OR longitudinal OR cohort OR usability
2. ( mhealth or mobile health or m-health or ) OR phone OR patient monitoring device OR personal digital assistan# OR wireless device# OR ( ehealth or e-health ) OR assistive technolog# OR smart technolog# OR smart device# OR platform OR app OR internet
3. p#ediatric OR child# OR ( adolesc# or teen# ) OR young pe#
4. juvenile idiopathic arthritis OR juvenile chronic arthritis OR juvenile rheumatoid arthritis OR jia

## **Search strategy using the CINAHL database**


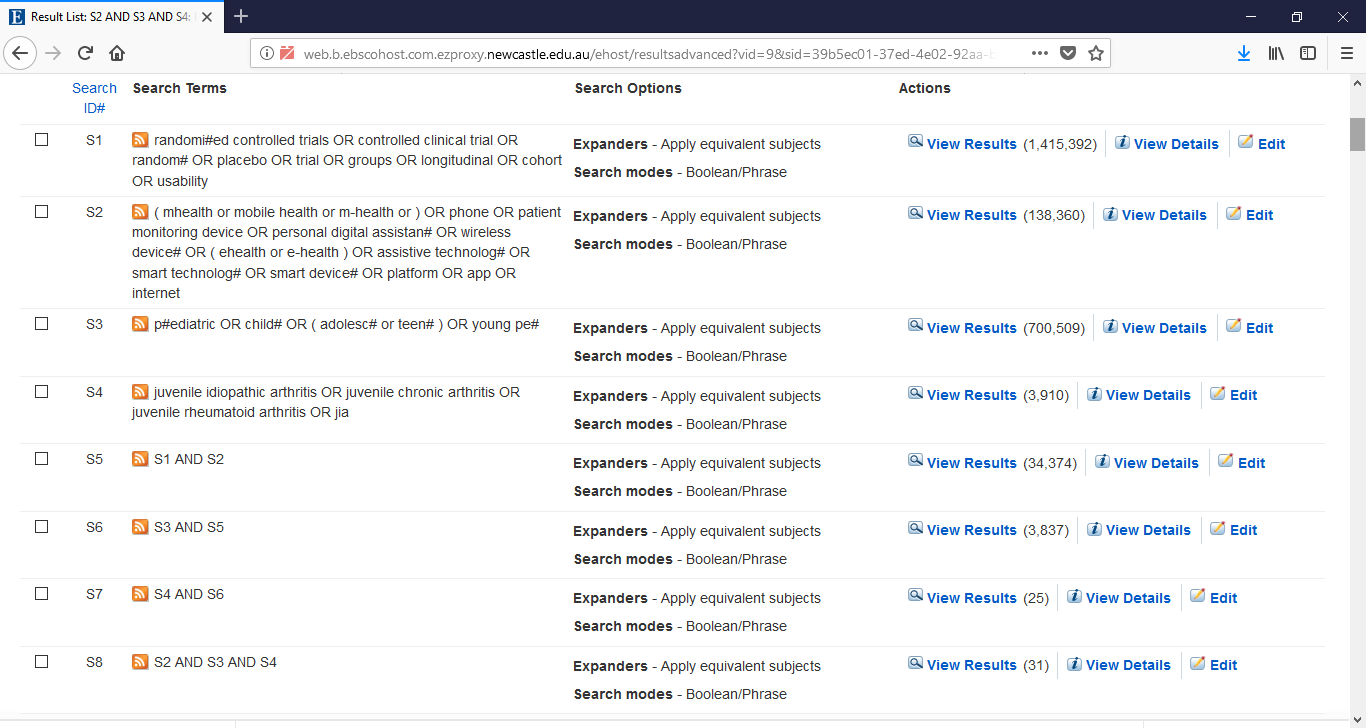

Supplement: Multimedia Appendix 2 [file pediatrics_v3i2e15833_app2.docx]
