# Supplementary material for: Usability of eHealth and Mobile Health Interventions by Young People Living With Juvenile Idiopathic Arthritis: Systematic Review
Source: JMIR Pediatr Parent. 2020 Dec 1;3(2):e15833. doi: 10.2196/15833 (PMC7738264; doi:10.2196/15833)
Supplement: Multimedia Appendix 3 [file pediatrics_v3i2e15833_app3.docx]

### Multimedia Appendix: Overview of the eleven eHealth and mHealth studies targeting Juvenile Idiopathic Arthritis

| First Author, Year,  Country | Sample size, age range | Intervention | Control | Outcomes | Study Design | DO |
| --- | --- | --- | --- | --- | --- | --- |
|  |  |  |  |  |  |  |
| Heale 2018,  Canada [59] | n=31, 12.8-18.6 years | Misfit Flash^TM^ | N/A | Physical activity | Pre and post intervention design;  Feasibility | n=3 |
| Armbrust 2017,  Netherlands [68] | n=49^a^,  8.7-10.8 years | Rheumates@Work^TM^ | n=21 | Physical activity | Multi-centre observer blinded RCT; Effectiveness | n=6^b^ n=1^c^ |
| Stinson 2016,  Canada [60] | n=39,  12-17 years | i*P*eer2Peer Program^TM^ | n=14^d^ | Peer mentoring | Pilot RCT; Feasibility, usability, effectiveness | n=8^b^  n=1^c^ |
| Armbrust 2015,  Netherlands [61] | n=83,  8-13 years | Rheumates@Work^TM^ | N/A | Self-management behaviour | Descriptive design;  Feasibility, usability | n=19 |
| Stinson 2014,  Canada [69] | n=70^a^  --^e^ | eOuch^TM^ | N/A | Pain | Correlational research,  Feasibility | --^e^ |
| Haverman 2013,  Netherlands [62] | n=176, Mean 11.6, SD 4.5^f^ | ePROfile^TM^ | n=67 | HRQoL | Sequential cohort study;  Effectiveness | n=0 |
| Stinson 2012, Canada [63] | n=101, 4-18 years | SUPER-KIDZ^TM^ | N/A | Pain | Descriptive design; Efficiency | n=4^g^ |
| Lelieveld 2010, Netherlands [64] | n=33,  8-12 years | Rheumates@Work^TM^ | n=16 | Physical activity | Pilot RCT;  Effectiveness | n=0 |
| Stinson et al. 2010, Canada [65] | n=46^h^, 12-18 years | Teens Taking Charge: Managing Arthritis Online^TM^ | n=24 | Disease management | Pilot RCT; Feasibility | n=4^b^ n=5^c^ |
| Stinson 2008, Canada [66] | n=13, 9-18 years | eOuch^TM^ | N/A | Pain | Descriptive  Study;  Feasibility, usability | n=3^i^ |
| Stinson 2008,  Canada [67] | n=112, 9-17 years | eOuch^TM^ | N/A | Pain | Prospective descriptive study; Feasibility, usability | n=2 |

Abbreviations: DO (dropout), N/A (not applicable).

1. Participants sample is the same as another study also included in this review
2. Intervention group
3. Control group
4. Wait list control
5. Not reported
6. Age range in years not available
7. Excluded from analysis (n=4, 4-7 years) due to parents completing pain assessment
8. Final analysis remained at n=46
9. Dropout replaced n=3 in phase 2

This is a Multimedia Appendix to a full manuscript published in the JMIR Pediatr Parent. For full copyright and citation information see http://dx.doi.org/10.2196/jmir.15833
