# Supplementary material for: Usability of eHealth and Mobile Health Interventions by Young People Living With Juvenile Idiopathic Arthritis: Systematic Review
Source: JMIR Pediatr Parent. 2020 Dec 1;3(2):e15833. doi: 10.2196/15833 (PMC7738264; doi:10.2196/15833)
Supplement: Multimedia Appendix 4 [file pediatrics_v3i2e15833_app4.docx]

**Overview of the seven eHealth and mHealth interventions for JIA**

| Name | Key features of intervention | Design descriptions | Data back up and security | Content alignment with policy, guidelines or validated tools | Training | Additional healthcare  support | Expected level of engagement |
| --- | --- | --- | --- | --- | --- | --- | --- |
|  | |  |  |  |  |  |  |
| **Misfit Flash^TM^** [59] | |  |  |  |  |  |  |
|  | Wearable activity tracker: feedback on activity levels, progress towards daily goals | Commercially available | Participants compatible smart phone |  | Verbal and written instruction | Research staff setup – synchronised watch to participants phone | 24 hours a day, 7 days a week, for 28 days |
|  | Young people: 12-18 years set daily goals | 12 month battery life. Water resistant to 30 metres |  |  |  |  |  |
|  | Measures step count, activity level, expended calories |  |  |  |  |  |  |
| **Rheumates@Work^TM^** [61,64,68] | | |  |  |  |  |  |
|  | Web-based educational program: identifying modifiable determinates of physical activity | Computer with internet access | Secure website: personal log in | Pender’s Health Promotion Model | 15 minutes: instructions on completing exercise diary, different PA categories and learning modules | Part-time staff, physician/psychologist to run 4 face-to-face group session; peers to share experience and model behaviour [47,49] | 1-3 hours a week for 14 to 17 weeks |
|  | Young people: 8-13 years set personal goals | Linear program design: Different theme each week. | Personal web page containing participants’ current level of PA, fitness and joint status | Cognitive behavioural and instructional model |  | Encourage to email questions to "Buddy", a cartoon |  |
|  | Interactive content: films, animations, spoken texts, puzzles, brain twisters, assignments | Feedback loop in program: verifies completion of activities or a reminder email sent |  |  |  | Progress monitored by administrator, email and reminder phone calls; safety monitored by PR |  |
|  | Program supported continuing activity during relapse |  |  |  |  | Chat sessions |  |
| **ePROfile**^TM^ [62] | |  |  |  |  |  |  |
|  | Web-based HRQoL questionnaire | Web based application - KLIK Website | Secure website: unique username and password | Preschool Children Quality of Life (TAPQOL) parent-reported for children (0-5years) | PR training | Patient’s answers  retrieved by PR during consultation | At home or before PR consultation |
|  | Self-reporting HRQoL by young people: 8-18 yrs, or by parents of children 0-7yrs | Answers tabulated to an *ePROfile^TM^* and colour coded to highlight concerns | PR can only view their own patients | Paediatric Quality of Life (PedsQL) Generic Core Scale (children 6-18 years and parent form) |  |  |  |
|  | Questionnaire: HRQoL, disease-specific information and functional ability |  |  | Childhood Health Assessment Questionnaire (CHAQ) |  |  |  |
|  |  |  |  | VAS 100mm for pain |  |  |  |
| **iPeer2Peer Program**^TM^ [60] | | |  |  |  |  |  |
|  | Online peer to peer mentoring program | Computer compatible with commercially available Skype software and internet access | Secure web-based server: video calls audio-recorded and uploaded | __ | Peer mentors, 2.5 hours on-site training. | All calls reviewed within 24 hours by research team to ensure safety | 10 times over 8 weeks for 20 minutes |
|  | Trained peer mentors (16–25 years, successfully managing their JIA), sex matched to participants (12–18 years) |  |  |  | Training related to the protocol prompting conversation and flagging concerns |  | Mean length of call 17.3 minutes/week |
|  | Topics of conversation not dictated by a protocol. Open ended and tailored questions to allow expression of needs |  |  |  | Instruction manual |  |  |
| **eOuch**^TM^ [66,67,69] | |  |  |  |  |  |  |
|  | Electronic pain diary with wireless capacity | Commercially available personal digital assistant (PDA): Tungsten series W: (Palm Inc, USA) | Data was encrypted in case the PDA was lost or stolen | Brief Pain  Inventory (modified) | 15 to 20 minutes, at home or clinic | 24 hr telephone support for problems (via a pager) | 3 times a day |
|  | Young people (9-18 years) used the Electronic VAS to self-report pain intensity, unpleasantness, and interference | Custom 5cm VAS designed for screen size. VAS then converted to 100-point scale | Data backed on memory card and/or uploaded (by cellular telephone network/wireless) to research centre, stored in study database or Microsoft Access (Microsoft Corporation, Canada) database |  | Demonstrated how to use PDA and software, using standardised pain vignettes | A phone call on day 1, day 7 and day 14 |  |
|  | Signal contingent approach: an audible alarm reminded users three times a day | Custom design software program: GraalPad (AppForge Inc USA) | Data dated and time stamped |  | eOuch^TM^ instruction manual |  |  |
|  | A compliance window of 30 minute to reply | All functions locked (ie calendars, internet browsing) | Data only accessible by the central research office computer |  |  |  |  |
| **SUPER-KIDZ**^TM^ [63] | |  |  |  |  |  |  |
|  | Web-based self-reporting pain assessment | SUPERKIDZ  Website, electronic device with  internet access | Secure website: participant account | Dephi technique to determined pain assessment domains | Training < 5 mins for participants and parent. Aim: to complete pain assessments without help from parents | Research assistant present to ensure participants completed assessment by themselves | Before consultation |
|  | Pain assessment domains: intensity, location, frequency, duration; and the consequence of pain. | Three devices compared: paper,  Apple iPod touch (2^nd^ generation)^TM^ 8 GB flash drive, 3.5 (diagonal) multi-touch display, laptop/computer- Internet Explorer 7.0 |  | Delphi study participants: CARRA^a^ members: PR and allied heatlh, Phase 1 n=115, Phase 2 n=157); and Consensus Conference (pain experts n=8, PR n=6, consumers n=3, allied health n=3) | Training on reasons for the assessment (i.e. pain), functionality, how to complete the assessments using each device |  |  |
|  | Children (4-7 years) used the Face Pain Scale Revised.  Adolescents: (13.5 years, SD 3.1 ) used 0-10 numerial scale | Linear program design: one question displayed at a time. Unaswered question reappear at the end. | To prevent data loss: user presses next to move to next question | Domains related to the predictive factors of chronic pain and pain-related disability | Training on how to reset the device in case of technical issues |  |  |
| **Teens Taking Charge: Managing Arthritis Online**^TM^ [65] | | | |  |  |  |  |
|  | Web-based program: disease specific information, management strategies and social support | Computer with internet access | Secure website: requiring log in | __ | __ | Telephone support by a trained coach, using a standardised script | 20-30 minutes each week for 12 weeks |
|  | Young people (12-18 years) set personal goals | Linear program design: 12 modules (1 per week) | Parents able to view the materials on the website | Goals tracked in “My Journal.” |  | Email, discussion board monitored daily |  |
|  | Two parents modules to encourage healthy behaviour and to help them “let go” | Tracks usage patterns |  |  |  | Unresolved problems referred to rheumatology team |  |

a. Childhood Arthrits and Rhuemotoly Research Alliance (CARRA)

This is a Multimedia Appendix to a full manuscript published in the JMIR Pediatr Parent. For full copyright and citation information see http://dx.doi.org/10.2196/jmir.15833
