# Supplementary material for: Usability of eHealth and Mobile Health Interventions by Young People Living With Juvenile Idiopathic Arthritis: Systematic Review
Source: JMIR Pediatr Parent. 2020 Dec 1;3(2):e15833. doi: 10.2196/15833 (PMC7738264; doi:10.2196/15833)
Supplement: Multimedia Appendix 5 [file pediatrics_v3i2e15833_app5.docx]

**Methodological scores of the eleven studies using the Down and Black (modified) checklist**

| Study author, year | Reporting | External Validity | Internal Validity^a^ | Internal Validity^b^ | Power | Total score | Judgement |
| --- | --- | --- | --- | --- | --- | --- | --- |
| Score  range | 0-11 | 0-3 | 0-7 | 0-6 | 0-1 | 28^c^ |  |
| Heale et al., 2018 [59] | 9 | 1 | 3 | 4 | 0 | 17 | Fair |
| Armbrust et al., 2017  [68] | 10 | 1 | 4 | 6 | 0 | 21 | Good |
| Stinson et al., 2016  [60] | 9 | 1 | 5 | 4 | 0 | 19 | Good |
| Armbrust et al, 2015  [61] | 9 | 3 | 4 | 3 | 0 | 19 | Good |
| Stinson et al., 2014  [69] | 8 | 1 | 4 | 2 | 0 | 15 | Fair |
| Haverman et al., 2013  [62] | 8 | 3 | 5 | 2 | 0 | 18 | Fair |
| Stinson et al., 2012  [63] | 8 | 2 | 4 | 4 | 0 | 18 | Fair |
| Lelieveld et al., 2010  [64] | 11 | 2 | 5 | 3 | 0 | 21 | Good |
| Stinson et al., 2010  [65] | 8 | 3 | 4 | 6 | 0 | 21 | Good |
| Stinson et al., 2008 [66] | 8 | 1 | 4 | 4 | 0 | 17 | Fair |
| Stinson et al., 2008 [67] | 9 | 2 | 4 | 3 | 1 | 19 | Good |

1. Bias
2. Confounding / selection bias
3. Total scores: Excellent: 24-28, good: 19-23, fair: 14-18 fair, poor: less than 14 [56]

This is a Multimedia Appendix to a full manuscript published in the JMIR Pediatr Parent. For full copyright and citation information see http://dx.doi.org/10.2196/jmir.15833
