# Supplementary material for: Usability of eHealth and Mobile Health Interventions by Young People Living With Juvenile Idiopathic Arthritis: Systematic Review
Source: JMIR Pediatr Parent. 2020 Dec 1;3(2):e15833. doi: 10.2196/15833 (PMC7738264; doi:10.2196/15833)
Supplement: Multimedia Appendix 6 [file pediatrics_v3i2e15833_app6.docx]

**Intervention adherence rates, including week to week, time of day and content adherence by young people with JIA**

|  | Misfit flash [59] | Rheumates@Work [61] | Rheumates@Work [64] | iPeer2Peer Program [60] | eOuch [69] | eOuch [66] | eOuch [67] | Teens Taking Charge: Managing Arthritis Online [65] |
| --- | --- | --- | --- | --- | --- | --- | --- | --- |
|  | | | |  |  |  |  |  |
| Adherence rates % | | | |  |  |  |  |  |
| Pre-specified timeframe | 72^a^ | 82.1 | 82 | 32 | 78 | Phase 1: 73  Phase 2: 70 | Study 1: 78  Study 2: 73 | N/A |
| Extended timeframe | N/A | 93.8 | 84 | 55 | N/A | N/A | N/A | 100^b^ |
| Weekly adherence % | | |  |  |  |  |  |  |
| Week 1 |  |  |  |  | 93 | Phase 1: 79  Phase 2: 75 | Study 1: 88  Study 2: 77 |  |
| Week 2 |  |  |  |  | 92 | Phase 1: 68, *P*<.05  Phase 2: 66, NS | Study 1: 73, *P*<.01  Study 2: 77 |  |
| Week 3 |  |  |  |  |  |  | Study 2: 67, *P*<.01 |  |
| Time of day adherence % | | |  |  |  |  |  |  |
| Morning – on waking |  |  |  |  |  | Phase 1: 84  Phase 2: 10% | Study 1: 84, *P*<.01  Study 2: 77, *P*<.01 |  |
| After school |  |  |  |  |  | Phase 1: 72, *P*<.05  Phase 2: 10% increase | Study 1: 73, *P*<.01  Study 2: 67, *P*<.01 |  |
| Evening – before bed |  |  |  |  |  | Phase 1: 71, *P*<.05  Phase 2: 10% increase | Study 1 and 2^c^  *P*<.01 |  |
| Content adherence: most common topics raised for discussion or learning modules visited | | | | | | |  |  |
| Top 1 |  | Arthritis |  | Management –  life-style^d^ |  |  |  | Arthritis |
| Top 2 |  | Management – pain |  | Arthritis |  |  |  | Management - symptoms |
| Top 3 |  | Management - energy |  | Management -pharmacological |  |  |  | Management - pharmacological |
| Top 4 |  | Setting goals |  | School/social  (non-JIA related) |  |  |  | Other types of care |
| Top 5 |  | Physical activity |  | Future concerns |  |  |  | Relaxation |

Abbreviations: (N/A) not reported, (NS) not significant – some NS results did not provided supporting statistics

a. Only 19% of participants wore their activity tracker 24 day/7 days a week/28 days, as per protocol [59].

b. Average length to complete the 12 week program, 14.7 weeks, range 12 to 21 weeks [67].

c. No adherence rates provided

d. Sleep hygiene, time management, staying motivated, following clinicians’ recommendations

This is a Multimedia Appendix to a full manuscript published in the JMIR Pediatr Parent. For full copyright and citation information see http://dx.doi.org/10.2196/jmir.15833
