# Supplementary material for: Usability of eHealth and Mobile Health Interventions by Young People Living With Juvenile Idiopathic Arthritis: Systematic Review
Source: JMIR Pediatr Parent. 2020 Dec 1;3(2):e15833. doi: 10.2196/15833 (PMC7738264; doi:10.2196/15833)
Supplement: Multimedia Appendix 7 [file pediatrics_v3i2e15833_app7.docx]

**Summary of the technical problems experienced by users with JIA, obstructing the engagement of the intervention**

|  | Technical problems |
| --- | --- |
|  |  |
| Misfit Flash^TM^ [59] | |
|  | 53% device malfunction rate |
|  | 29% flat battery (expected battery life - 12 months) |
|  | 13% stopped working due to being worn in the water (only water resistant) |
|  | Activity tracker discs fell out of the wristband (n=3) |
| Rheumates@Work^TM^ [61] | |
|  | 2.7% (mean 1.7) of participants each week: forgotten login details, incorrect colouring, unable to print assignment |
| SUPERKIDZ^TM^ [63] | |
|  | Weak wireless network signal: delayed and missed responses in the data base due to using iPod and computer |
| Teens Taking Charge: Managing Arthritis Online^TM^ [65] | |
|  | Log in problems for website, resulted in n=2 drop out |
| eOuch^TM^ [66,67,69] | |
|  | 22% of data missing on the database, despite good adherence |
|  | Software problem: PDA digitizer sensitivity caused skipping to next question without the participant tapping the screen. |
|  | Freezing occurred when sending data, when wireless network was unavailable |
|  | Wireless network unavailable for a period of 8 to 10 hours |
|  | Creation of an additional email gateway occurred without notification |
|  | Accidental cancellation of one participants wireless plan |

This is a Multimedia Appendix to a full manuscript published in the JMIR Pediatr Parent. For full copyright and citation information see http://dx.doi.org/10.2196/jmir.15833
