# Supplementary material for: Usability of eHealth and Mobile Health Interventions by Young People Living With Juvenile Idiopathic Arthritis: Systematic Review
Source: JMIR Pediatr Parent. 2020 Dec 1;3(2):e15833. doi: 10.2196/15833 (PMC7738264; doi:10.2196/15833)
Supplement: Multimedia Appendix 8 [file pediatrics_v3i2e15833_app8.docx]

**User feedback: Results of Acceptability and Satisfaction questionnaires**

|  | Usability, Acceptability, Satisfaction | Features liked | Features disliked, improvements |
| --- | --- | --- | --- |
|  | |  |  |
| **Misfit Flash^TM^** [59] | |  |  |
|  | Enjoyed (27/28, 96%) | Liked being physically active (20/28, 71%) | No feasibility/usability questionnaire used to gained feedback |
|  | Expressed an interest in purchasing an activity tracker after the study (25/28, 89%) | Felt activity increased as a result of the intervention |  |
| **Rheumates@Work^TM^** [61] | |  |  |
|  | Little or no encouragement by their parents (46/64, 71%) | Liked (participants 52/64, 81%, parents 63/64, 99%); liked making new friends and Buddy | No specific exercise instruction, more detailed exercise program needed, more physical assessments |
|  | Parents rarely or never help their children (48/64, 75%) | Understood the topics well or very well (61/64, 95%) | Target age (8 -13 years) too broad; content slightly difficult for the younger participants, too childish for the older participants. |
|  | Program/assignments adequate or too easy (57/64, 89% - 62/64, 97%) | Learnt something, or quite a lot (participants 54/64, 85%, parents 48/64, 75%) | One participant withdrew due to program being too difficult |
|  | Time invested was fine or too short (53/64, 83%) |  | Buddy (the email character) was not original. |
|  |  |  | Chat sessions (17/64, 27% adherence) |
| **ePROfile^TM^** [62] | |  |  |
|  | Parents’ median evaluation score 8/10 (range 4-10) | Parents reported:  usefulness (t1^a^=57/65, 88%, t2^b^=37/46, 80%), helpfulness (57%), and helpfulness for their child (60%). | Parent satisfaction did not differ between IG & CG. Child (mean age 11.6 SD 4.5) satisfaction not assessed. |
|  | Parents reported: reflected their child’s HRQoL adequately (t1=61/65, 94%, t2=42/46, 91%) |  | Children reported the PR consultation using *ePROfile^TM^* as normal (47/48, t1=98%, t2= 29/35, 83%)^a^ |
| **iPeer2Peer Program^TM^** [60] | |  |  |
|  | All adolescents reported satisfaction | Liked meeting someone with JIA they could relate to; someone older who has already experienced what they are going through (JIA and non-JIA related); having someone to talk to. | May be beneficial targeting specific support needs. For example, social support, challenging symptoms or active disease |
|  | Would recommend to others | Liked getting information about JIA |  |
| **eOuch^TM^** [66,67] | |  |  |
|  | Quick and easy to use, easy to complete three times a day over 2-3 weeks | Liked how pain diary looked |  |
|  | Willing to use more than 2-3 weeks (>50%) | Not bothersome, minimally interfering with activities and friends |  |
| **SUPER-KIDZ^TM^** [63] | |  |  |
|  | Acceptability using paper, computer and an Apple iPod touch to record pain: Adolescents (8-18 years) and parents reported no difference. | Adolescents: computer or paper more useful for describing pain than the iPod (60/77, 78%, *P*=.03) | Adolescents: least liked iPod (42/77, 54%, *P*<.001) due to: size, unfamiliarity, increased time taken to register responses |
|  | Parents reported children (4-7 years) preferred the computer (16/24, 65%, *P*=.008) because most simple, and fun to use | Parents: computer or paper assessments easier to understand than the iPod (21/24, 91%, *P*=.03) | Adolescents: paper the most inappropriate device for their age group (65/77, 84%, *P*=.004) |
|  |  | Adolescents (67/77, 87%, *P*<.001), parents (21/24, 91%, *P*=.02) perceived computer or paper to be quicker |  |
| **Teens Taking Charge: Managing Arthritis Online** [65] | | |  |
|  | Easy to use and highly acceptable | Liked: content, videos, audio features (relaxation and guided imagery), and personalisation through interactive features. | Parent engagement in parent modules low |
|  | Would recommend to others | Felt the coach was an essential component |  |

Abbreviation: NS (not significant)

a. First consultation (t1), second consultation (t2)

This is a Multimedia Appendix to a full manuscript published in the JMIR Pediatr Parent. For full copyright and citation information see http://dx.doi.org/10.2196/jmir.15833
